# Mixed‐methods exploration of views on choice in a university asymptomatic COVID‐19 testing programme

**DOI:** 10.1111/bioe.13012

**Published:** 2022-02-28

**Authors:** Caitríona Cox, Akbar Ansari, Meredith McLaughlin, Jan W. van der Scheer, Jennifer Bousfield, Jenny George, Brandi Leach, Sarah Parkinson, Mary Dixon‐Woods

**Affiliations:** ^1^ THIS Institute Clifford Allbutt Building Cambridge Biomedical Campus Cambridge UK; ^2^ Homerton College Homerton College Cambridge UK; ^3^ RAND Europe Westbrook Centre Cambridge UK

**Keywords:** bioethics, COVID‐19, education, mixed‐methods, public health, testing

## Abstract

Asymptomatic COVID‐19 testing programmes are being introduced in higher education institutions, but stakeholder views regarding the acceptability of mandating or incentivizing participation remain little understood. A mixed‐method study (semi‐structured interviews and a survey including open and closed questions) was undertaken in a case study university with a student testing programme. Survey data were analysed descriptively; analysis for interviews was based on the framework method. Two hundred and thirty‐nine people participated in the study: 213 in the survey (189 students, 24 staff), and 26 in interviews (19 students, 7 staff). There was majority (62%) but not universal support for voluntary participation, with a range of concerns expressed about the potentially negative effects of mandating testing. Those who supported mandatory testing tended to do so on the grounds that it would protect others. There was also majority (64%) opposition to penalties for refusing to test. Views on restricting access to face‐to‐face teaching for non‐participants were polarized. Three‐quarters (75%) supported incentives, though there were some concerns about effectiveness and unintended consequences. Participants emphasized the importance of communication about the potential benefits of testing. Preserving the voluntariness of participation in student asymptomatic testing programmes is likely to be the most ethically sound policy unless circumstances change.

## 1 INTRODUCTION

**FIGURE 1:**
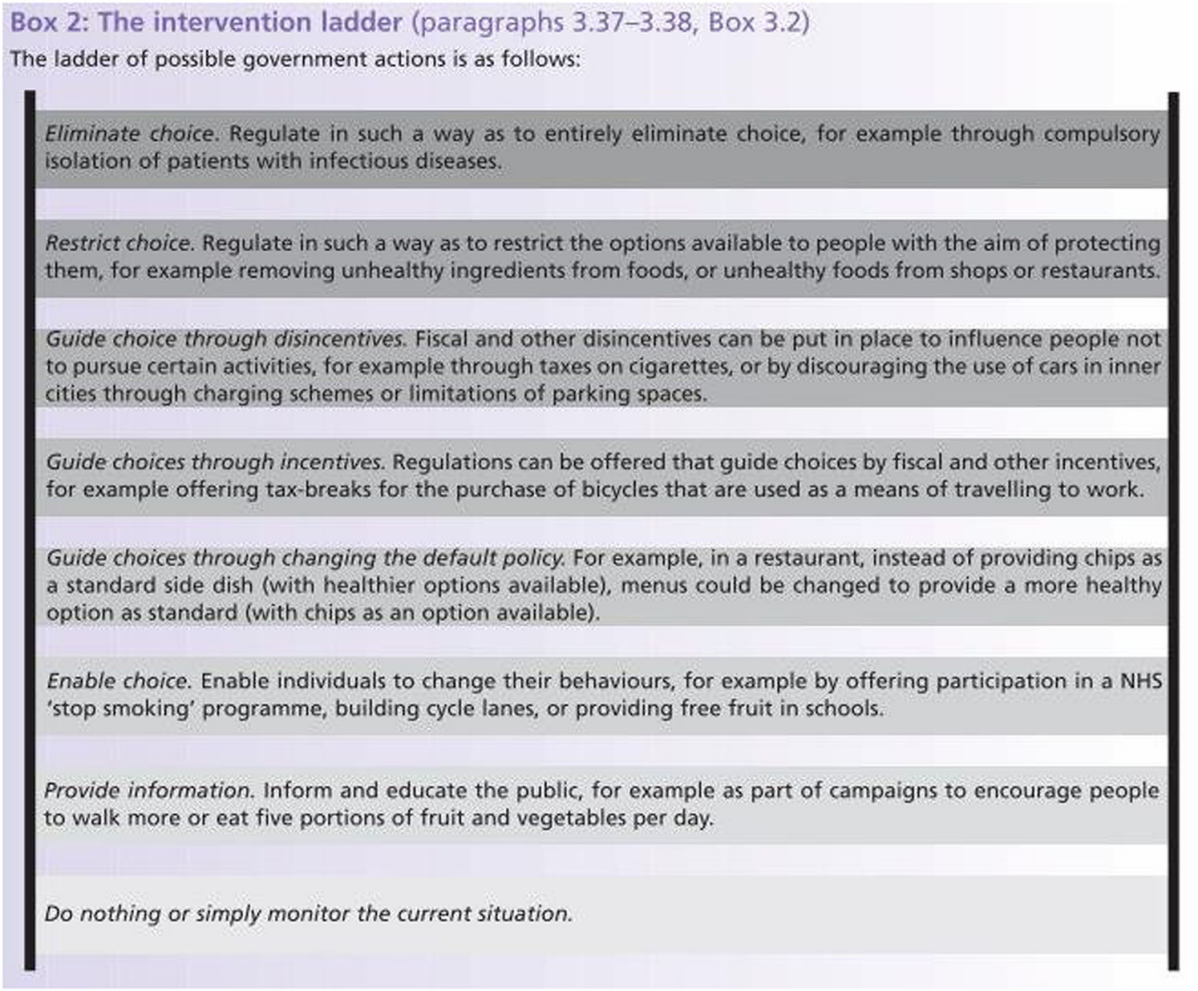
The intervention ladder, reproduced with permission from the Nuffield Council of Bioethics report

A widely acknowledged challenge in public health ethics is the need to balance individual autonomy and restrictive actions for the collective good, for example when coercive policies are proposed to protect and promote the health of others.^1^ A principle common to many of the frameworks seeking to address this tension is that public health interventions should interfere with the autonomous freedom of individuals to the least possible or necessary extent.^2^ This principle of *least restrictive means* is practically embodied in the Nuffield Council of Bioethics’ 2007 ‘intervention ladder’ (Figure 1), according to which public health interventions should ideally infringe on individual liberty minimally, with movement ‘up’ the ladder—towards more restrictive measures—requiring increasingly greater justification.^3^ For instance, the ladder suggests that incentives should be used in preference to penalties.

Discussions about the justifiability of restrictive public health measures are particularly salient in the context of infectious diseases, which raise distinctive ethical issues by virtue of their communicability.^4^ There is a significant difference between interventions designed to control individuals’ behaviours for their own well‐being (for example those aimed at reducing risk for coronary heart disease) and those that limit the autonomy of individuals in order to control the spread of harmful disease to others.^5^ Restrictive infectious disease control measures, such as isolation and quarantine, are classically justified using Mill's harm principle, according to which they are acceptable as they prevent harm to others.^6^

The COVID‐19 pandemic has brought many of these issues vividly into view. Measures to control transmission—including case isolation, household quarantine, and contact tracing—have been described by Gostin as among ‘the most complex and legally/ethically controversial public health powers’.^7^ An important feature of the response to COVID‐19 has been the introduction of mass testing of asymptomatic individuals, with subsequent isolation of individuals who test positive (and their close contacts). This has been implemented in a range of settings in the UK and elsewhere, but has been controversial. Some of the criticism has focused on questions about the effectiveness of mass testing in reducing COVID‐19 transmission,^8^ linked to lack of supporting evidence.^9^ Others have raised concerns relating to cost^10^ and the potential harms associated with false negatives.^11^ In higher education settings, where testing has been introduced on a large scale, several of these concerns have been raised specifically.^12^

Recent UK Government guidance suggests that higher education institutions can consider incentives for compliance, and disincentives for non‐compliance, to enforce COVID‐19 public health measures.^13^ There is, however, limited evidence about the views of students or staff on these measures. The need for a participatory approach has been emphasized in recent work in empirical ethics, according to which empirical methods can be integrated with ethical analysis to address normative issues.^14^ Explicit consideration of how stakeholders might help to guide the development of public health interventions has also been highlighted in a number of public health ethical frameworks.^15^ In this context, the need for research into stakeholder views on normative issues relating to asymptomatic COVID‐19 testing programmes in higher education institutions—such as the ethical acceptability of different policies to encourage participation—is clear.

In this paper, we report a study of the views of staff and students on choice relating to participation in a student testing programme at a case study university. We sought to examine in particular the acceptability of mandating participation in testing programmes, and the extent to which options that stop short of mandating, such as incentives or penalties, might be seen as legitimate.

## 2 METHODS

The data presented here were collected as part of a wider project on the ethical issues in asymptomatic testing programmes for students in higher education.^16^

### 2.1 Study context

The case‐study institution, the University of Cambridge, introduced a programme involving weekly asymptomatic pooled COVID‐19 testing of students in October 2020. It is a collegiate university, made up of 31 colleges, which are separate institutions affiliated with the university. The colleges are responsible for providing students with accommodation and catering facilities, and academic and pastoral support. Testing was offered to all students living in college‐owned accommodation in October 2020. Its programme was based on obtaining nasal swabs, which were tested by polymerase chain reaction (PCR) in university laboratories. Testing was based around ‘households’ (typically consisting of 8–10 students, often arranged around a shared kitchen or bathroom), with nasal swabs from individual students all being tested in a pool. If a pooled sample tested positive, students within the household were asked to isolate immediately. Confirmatory individual testing by PCR then took place. If any of the students within the household tested positive on this confirmatory testing, all in the household were required to isolate for 14 days.

At the time of our study, the testing programme was available to all students living in college‐owned accommodation and participation was voluntary for individual students; there was no incentivization or penalization surrounding participation in the testing programme. It is important to note that this study took place before COVID‐19 vaccination was widely available, and the results should be interpreted in this context.

### 2.2 Study design and data collection

The study used a mixed‐method approach (semi‐structured interviews and an online survey including Likert‐scale and open‐ended questions) to gather views of students and staff. We began by developing a provisional ethical framework, drawing on a previously developed example in the area of testing of healthcare staff,^17^ and a review of relevant literatures on public health, ethics and screening. This guided the development of our study instruments and initial analysis of the findings.

We used mass emailing lists to invite students (undergraduate and postgraduate) to participate. Staff (both academic and non‐academic) were invited, also using email, by the colleges in which they worked. Eligible participants were over 18 years old, able to understand and speak English, and were either currently registered University of Cambridge students (any subject or year of study) or a member of staff currently employed by the University of Cambridge or a college.

Emails included full details of the project and an explanation of how to take part. Interested people were asked to register on Thiscovery (https://www.thiscovery.org/about), an online research and development platform created and developed by THIS Institute at the University of Cambridge. They were able to choose to participate in an interview or complete an online survey. All participants provided consent prior to the interview or survey.

Data were collected over a single period running from November 20 to December 11, 2020. The survey was administered using Qualtrics (Supporting Information Appendix S1), and took approximately 15 min to complete. It contained a mixture of closed, open and Likert‐scale questions. An initial version of the survey was developed and piloted with 10 students in order to refine the questions. Only minor changes were made after piloting, which involved altering the wording of questions to make them more understandable to participants.

Semi‐structured interviews took place either online using audio‐video software or by telephone. Interviews lasted 30–60 min and were conducted using a prompt guide (Supporting Information Appendix S2). The prompt guide was refined through pilot interviews with three students; as with the survey, the main changes that were made after piloting were minor. They concerned the phrasing of questions to enhance clarity and the order of questions to enhance the flow of the interviews. The interviews were transcribed verbatim.

We did not undertake a formal test for theoretical saturation; we instead used the principle of ‘information power’, which indicated that we have achieved sufficient range and depth of views.^18^

### 2.3 Data analysis

The quantitative closed‐ended survey questions were analysed using descriptive statistics, and Likert‐scale questions were visualized using diverging stacked bar charts. Our analysis approach for the open‐ended survey responses and interview data was broadly based on the framework method. This method allowed multiple analysts to scrutinize the data, comparing views of participants to identify commonalities and divergences.

After familiarizing themselves with the data, two analysts independently coded the first three interview transcripts deductively, using pre‐defined codes based on the provisional ethical framework. A group of researchers then scrutinized the work of the two analysts to agree on a set of codes to apply to all transcripts, including an ‘other’ code to host data that did not fit any of the pre‐defined codes. A matrix was then produced by applying this coding framework to all transcripts, in which quotes were organized into codes and copied into an Excel spreadsheet. A summary was then written for each code, including references to interesting or illustrative quotes. Free‐text responses to survey questions were analysed in a similar manner, with the production of a separate matrix and coding summaries for these data.

We integrated the three data types—interview, survey open‐question responses and survey closed question responses—at the interpretation stage of analysis.^19^ We discussed patterns arising across our analyses of the three types of data through an iterative process that involved examining all the data for themes, and considering convergence or divergence between sources. Insights from interviews and the survey were given equal priority in the interpretation.

## 3 RESULTS

A total of 239 stakeholders participated in the study: 189 students and 24 staff completed surveys; 19 students and seven staff participated in semi‐structured interviews (Table 1). Overall, the interview and survey data did not significantly diverge in their findings, and the results below reflect insights from both datasets.

**TABLE 1:** Participant demographic information

Participant characteristic	Survey *N* (%)	Interview *N* (%)
Role		
College academic (e.g. fellow/tutor)	5 (2.3)	5 (19.2)
College staff	17 (8.0)	2 (7.7)
Other staff	2 (0.9)	0 (0.0)
Student (postgraduate)	56 (26.3)	8 (30.7)
Student (undergraduate)	133 (62.4)	11 (42.3)
Residence in college		
No	23 (10.8)	8 (30.6)
Yes	189 (88.7)	17 (65.4)
Sex		
Female	126 (59.1)	11 (42.3)
Male	85 (39.9)	15 (57.7)
Prefer not to say	2 (0.9)	0 (0.0)
Ethnicity		
White	171 (80.3)	20 (76.9)
Mixed/multiple ethnic groups	8 (3.8)	1 (3.8)
Asian/Asian British	26 (12.2)	2 (7.7)
Black/African/Caribbean	2 (0.9)	0 (0.0)
Arab	0 (0.0)	2 (7.7)
Other	2 (0.9)	0 (0.0)
Prefer not to say	4 (1.9)	1 (3.8)
Disability, illness or impairment causing difficulties with day‐to‐day activities		
No	184 (86.4)	23 (88.5)
Prefer not to say	5 (2.3)	2 (7.7)
Yes	24 (11.3)	1 (3.8)

We present our integrated findings under three themes: views on voluntariness of student participation in the testing programme, circumstances under which mandatory testing might be deemed ethically acceptable, and legitimacy of options for encouraging participation.

### 3.1 Views on voluntariness of participation in the student testing programme

Just under two‐thirds of survey respondents agreed (40% agreed, 22% strongly agreed) with the statement that asymptomatic testing should be voluntary at the level of the individual student (Table 2).

**TABLE 2:** Survey data regarding choices about testing

Statement	Strongly disagree	Disagree	Neutral	Agree	Strongly agree	*N*
Asymptomatic COVID‐19 testing should be voluntary at an individual student level: individual students should be allowed to opt out	23 (11%)	37 (17%)	21 (10%)	85 (40%)	47 (22%)	213
It is acceptable for colleges to incentivize students for taking part in asymptomatic COVID‐19 testing (for example by giving them a free coffee voucher)	8 (4%)	18 (9%)	27 (13%)	89 (42%)	70 (33%)	212
It is acceptable for colleges to penalize students who decline to take part in asymptomatic COVID‐19 testing	65 (31%)	71 (33%)	32 (15%)	31 (15%)	14 (7%)	213

In both free‐text survey responses and interviews, participants who supported voluntary testing cited reasons such as respect for personal liberty and individual choice. This was particularly true for those who conceptualized testing as a medical intervention involving a physically invasive test, one over which individuals should retain the right to choose. In these accounts, preserving individual choice was presented as the predominant ethical consideration. Even when participants strongly supported the view that students should participate in testing—for reasons such as protecting others in the community—some felt that it should still be up to the individual to decide.

Accepting a COVID test involves a physical process of taking swabs, meaning that it is extremely important that each student has the right to consent or decline consent to take part. (S_161_undergrad)

No, I don't think it would be acceptable [to mandate testing]… I think it would be rather a great infringement of personal rights… ultimately it should be your own choice. (I_1_undergrad)

Although I personally strongly believe everyone should take the test, I believe it would be a violation under most laws and moral frameworks to force them to take part. (S_1_undergrad)

In interviews, some students suggested that they personally would not have strong objections to the testing programme becoming mandatory, but they could envisage other students opposing it, or being more negatively impacted by a mandatory testing policy.

I mean, I personally don't think there is a problem with making it mandatory… but I suppose everyone's in a very different situation and maybe there are some unintended consequences… which might impact people's lives a bit differently. (I_7_postgrad)

Staff similarly indicated that they did not think mandatory testing would be accepted by the majority of the students, with some raising the question of whether it would be legally enforceable. In general, staff focused on the potential problems that might be associated with trying to introduce mandatory testing, with very few suggesting that it should be attempted.

Well, knowing our students, I don't think it could be made compulsory… We'd have protests and all the complaints if it was. (I_21_staff)

My understanding of the legal position here is that you cannot request it in order to be a student, but we could potentially request it both as a landlord and in safeguarding our staff. So, yeah, I would love to make it compulsory, but I think that would be a whole different kettle of fish in trying to enforce that. (I_16_staff)

Participants identified potential negative effects of making testing mandatory, for example the potential to change the character of the programme and produce hostility or resentment, even among students who were happy to voluntarily take part. The principle that students should still be able make a choice, even when their choice would have been to participate, was emphasized. One possible impact of mandating participation identified in interviews and survey free‐text responses was the risk that students might subvert the programme by spoiling their tests. For this reason, the usefulness of making a self‐administered test mandatory was questioned by some.

If it becomes compulsory, then it becomes a very different regime of you must come back to university and you must stick a stick up your nose every week, and it becomes a bit more authoritarian. (I_5_undergrad)

I don't think testing can be enforced on everyone. As students carry it out themselves, if they didn't want to take part but were forced to they just wouldn't do it properly. (S_201_staff)

### 3.2 Circumstances under which mandatory testing might be considered ethically acceptable

In the survey, 28% of participants felt that participation in the testing programme should be made mandatory for individual students (Table 2). In both interviews and the survey free‐text responses, this view was justified as a reasonable condition of living in college accommodation: on the basis of an obligation to act towards a common good, or to protect the safety of others within the college community. Parallels were drawn between mandatory testing and other accepted mandatory measures, such as mask‐wearing, or the existing requirement for healthcare students to have certain vaccines.

I realise it's a breach of personal liberties to enforce it on everyone, but I have no problem with enforced weekly tests being a precondition for living in college‐owned accommodation, where you are putting other students at risk if you don't cooperate. (S_102_undergrad)

I'd like to see it mandatory, just because I myself can't see a reason why you wouldn't want to… You know how masks are sort of you have to wear them unless you have an exemption, I'd like to see that policy introduced into the testing. (I_6_undergrad)

In interviews, we specifically asked participants if there were situations when they might consider mandatory testing acceptable, even if they did not support it currently. Some participants felt that it would never be. Others speculated that if levels of infection, or the COVID‐19 mortality/morbidity, were higher, mandatory testing might be acceptable. Other situations in which mandatory testing was seen as potentially acceptable were in controlling specific outbreaks, in households with a clinically vulnerable person, or in situations when students were about to travel home for the holidays.

I think if COVID was a much, much worse disease particularly for young people then there would be an argument there… If it was something where if you get it you'd definitely die or have a severe reaction then, yes, I'd be a bit more comfortable with them forcing students. (I_13_postgrad)

### 3.3 Legitimacy of options for encouraging participation

Around two‐thirds of survey respondents demonstrated opposition to the idea of penalties for those who declined to participate in the testing programme: 31% strongly disagreed, and 33% disagreed, that it is acceptable for colleges to penalize students for not taking part (Table 2). In survey free‐text responses and interviews, some viewed limited forms of restriction as acceptable, but many saw a punishment‐focused approach as undermining choice and risking creating feelings of resentment and hostility towards the programme.

It would be unacceptable to penalise students who do not feel comfortable to take part… any action which involves physically invasive processes should be entirely at the discretion of the individual. (S_161_undergrad)

I think punishing people for not taking part is less gainful than encouraging them to participate; however, some small measures, such as restriction of access to certain College areas if untested, might be considered a compromise. (S_205_postgrad)

Views were polarized on the idea of restricting access to face‐to‐face teaching for those who do not take part in the programme. Some expressed in interviews that it would be a reasonable and fair thing to do, because untested individuals who attend teaching in person could potentially endanger others (including more clinically vulnerable staff). Others suggested that students had a strong right to access the full possible range of educational opportunities because they paid tuition fees.

I personally would approve of [limiting access to in‐person teaching for non‐participating students] and I think it would have a positive impact on those that are attending the face‐to‐face lectures… there's that element of safety amongst the people that are attending… restricting educational opportunities, it kind of does ring alarm bells, but I'm not sure why people wouldn't do the testing programme really. So I wouldn't see a problem with it. (I_7_postgrad)

That's a tricky one because in a way it's punishing, it's a penalty but then it's for the safety of others as well. So, in that situation, your choice of not taking a test is not only affecting yourself but also others… but then if I think of my flatmate, she would go nuts if she found out she wouldn't be able to attend in‐person teaching if she doesn't take the test. (I_1_undergrad)

Some were strongly opposed to the idea, stating that it would be fundamentally wrong to inhibit access to teaching, suggesting that it would undermine the entire purpose of the university.

That would be very unethical. Or is that the right word? … If that were to ever come about, I'm sure the whole student body would cause an uproar… I would be very angry… it's an educational institution and that's their priority, is to make sure that you receive the good, if not excellent, education at this institution. And so, taking away one of the key things which makes this institution what it is would be kind of sacrificing such a valuable part that I don't know how they would ever be able to justify something like that. (I_14_undergrad)

Incentives were seen as more acceptable than penalties: most survey respondents supported their use (42% agreed, 33% strongly agreed; Table 2). In interviews, both staff and students saw small incentives (such as a free coffee) as acceptable. Distinctions were sometimes drawn between the use of small and large incentives; small incentives were seen as more acceptable. Some strongly welcomed incentives on the grounds of encouraging uptake and also ‘thanking’ participants for participating in the testing programme (which was conceptualized as an essentially altruistic act).

[I]t's just as a means of acknowledgement or saying that this is something that matters. So making people feel that they're… taking part and their contribution is very much welcomed… It doesn't even need to be a cup of coffee, to be honest, it could be like one sweet, I think, or something like that. (I_4_postgrad)

Thanking somebody for an altruistic act is absolutely fine. I don't think large bribes, so I don't think large amounts of funding would be appropriate, but I think a small gift as a thank you is similar to when I give blood. I get a biscuit and a cup of tea. That seems entirely fine and I don't consider that a bribe. (I_26_staff)

A minority of survey respondents (12%) opposed the use of incentives. Interview and survey free‐text data provided insight as to why: some suggested that small incentives might not be very effective in increasing uptake, particularly in students who already had concerns about the programme, and some had concerns that incentives might undermine choice and quality of consent.

Personally, I think that things like a free coffee… would not change most students' opinions who were on the cusp of trying to work out whether or not to do it or not. (I_16_staff)

I have a hard time agreeing with incentivising students to give consent to the asymptomatic testing because one can then question whether it is truly a consent. (S_106_undergrad)

Others described how offering incentives that might be perceived as menial could damage the programme by sending the wrong message about its value and trivialize its importance; instead, students might be more motivated by a desire to act for the good of the community than for a small tangible reward.

Yeah, but the problem with incentivising things, it then kind of undermines what you're trying to do… which I have a slight problem with… I just start thinking, okay, am I doing this really to actually make sure that I'm safe and I'm well, or is it just to get this coffee or whatever incentive? (I_14_undergrad)

A more general objection to use of either incentives or penalties was founded in the view that their use might be infantilizing and disrespectful to students’ adult and responsible status, expressing distrust in their ability to make sensible and informed decisions.

[T]hat's saying, well, there are prizes if you're good and there are punishments if you're bad, which I think is infantilising and wrong. (I_9_undergrad)

I don't think [students] want either positive or negative incentives, they just want to be asked. They're quite proud of that sort of, you know, here they are, adults; their adult status is really what they want more than anything else. (I_21_staff)

The importance of communication in maintaining trust in the testing programme was highlighted in interviews with both students and staff. Participants discussed that good education and persuasion would be a more effective and reasonable method of encouraging participation than introducing mandates or incentives.

So in terms of information to students, I think probably need to be as transparent as possible. I think if there's any feeling that they're not being given the whole picture, that will only lead to distrust and to cynicism. (I_19_staff)

[Y]our biggest weapon is actually providing good quality evidence. We're supposed to be educating young people to weigh up and argue whether in a scientific or an arts setting, providing them with quality information that demonstrates to them why we are saying that this is a good thing to do. And if you can produce that evidence and show them… I think you would be much more successful than if you tried to make it mandatory. (I_18_staff)

I think it's a matter of communication. If you communicate why this is important and why you're doing so much, why it's so much better, why there are so many better reasons for doing this than a coffee, then I think that should be enough. (I_23_staff)

## 4 DISCUSSION

Debates in public health ethics often identify the tensions between individual autonomy and restrictive actions for the collective good.^20^ Though sometimes over‐emphasized and over‐simplified,^21^ it is important that they are systematically considered in approaches to controlling infectious disease, where quarantine and isolation in particular ‘graphically illustrate the tension between individual right and collective good’.^22^ Many of these challenges were evident in our study of over 230 stakeholders in a COVID‐19 student testing programme in a case study university, which found broad support for voluntary participation, broad opposition to the use of penalties, and tentative support for use of incentives (tempered with some concerns over their effectiveness and their unintended consequences).

While many participants perceived an ethical obligation to take part in the testing programme (for example to protect others in the community), they also saw getting tested as a moral choice that should not be forced. Some felt that the obligation to protect others was sufficient to warrant compulsory testing, but the majority favoured an approach supporting individual autonomy in deciding whether to participate. Our study further suggests that effective communication may be the most legitimate and morally defensible approach to encouraging participation, and may be especially important while the evidence‐base for mass asymptomatic testing as a means of reducing transmission remains uncertain. Three major reasons for preserving the principle of least restrictive means for asymptomatic testing programmes in higher education institutions can be identified from this analysis, which relate to: *effectiveness*; *necessity*; and the *moral and symbolic implications* of enabling or limiting choice.

One reason for preserving voluntariness lies in the possible consequences of more coercive approaches on the *effectiveness* of securing participation in student testing programmes. Effectiveness—how far an intervention or programme delivers on its goals—is a key consideration in the design and operation of public health measures.^23^ In encouraging participation in a public health programme, it is important to critically consider whether directive measures are likely to be more effective than voluntary ones.^24^ Our study suggests that efforts to make programme participation mandatory, or to stimulate participation using penalties, could undermine effectiveness. For example, some participants suggested that such measures could provoke hostility towards the testing programme, perhaps, for example, reducing cooperation with correct self‐swabbing technique.

*Necessity*—in this case the extent to which a mandatory approach is needed—is also a relevant consideration. According to the principle of the least restrictive means, restrictive measures should only be used where less restrictive means have failed to achieve appropriate ends.^25^ This means that policy‐makers should not only consider whether a coercive approach is likely to be effective, but also whether it is essentially required. As Childress et al. note, the burden of moral proof lies with proponents of a forcible strategy: they must be able to justify the need for a coercive model above a voluntary one.^26^ We found widespread support for the testing programme—99% of survey respondents supported or strongly supported it.^27^ Furthermore, at the time of our study, participation in the programme was persistently >75% for eligible students.^28^ In this context, it is questionable whether a more restrictive approach is truly necessary for a student asymptomatic testing programme to reach its goal of minimizing viral transmission. Interventions that move up the intervention ladder (Figure 1)^29^ should only be considered if evidence emerges that participation in a testing programme is faltering, or if there is a material change in the wider situation that might shift risk‐benefit calculations (for example, the emergence of a new, more dangerous variant).

Yet our analysis goes beyond considerations of effectiveness or necessity, in drawing attention to the *moral and symbolic implications* of enabling or limiting choice in relation to testing. In public health interventions, Byskov suggests measuring the least restrictive means by considering whether certain normatively valuable capabilities are being restricted or protected.^30^ Consistent with some evidence from another higher education institution,^31^ most stakeholders in our study saw taking part in testing as the ‘right thing to do’, often citing the importance of reducing viral transmission to others (particularly vulnerable members of the community). Overall, these findings affirm Childress et al.'s position that often the most defensible approach to screening or testing ‘expresses community rather than imposes it’.^32^ For participants in our study, being able to choose to do good—rather than being forced, either through the programme being mandated or through threat of punishment—was an important form of respect for their moral agency. In a different but nonetheless relevant context, Brownsword worries that being denied the opportunity to choose to be good may undermine the conditions required for moral community, arguing that the opportunity to lead an authentic moral life takes us to the essence of human dignity.^33^ In a similar vein, Buchanan writes, ‘[o]ther things being equal, a society in which people choose to behave responsibly, rather than being forced against their will… is inherently more desirable’.^34^

Opposition to incentives was to some extent similarly grounded in their symbolic status: although the majority of participants supported their use, some worried they could potentially undermine the solidarity‐based values of the programme and encourage participation for the wrong reasons. Such views are in keeping with a broader conceptualization of participating in testing as an altruistic endeavour, which is intrinsically valuable for its contributions to public health and does not demand additional remuneration. Sandel has written about the potentially corrupting influence on valuable social norms of incentivizing previously non‐monetized practices.^35^ The corrosive diminishment of value associated with incentivizing health behaviours has been criticized for its lack of supporting empirical evidence.^36^ Although the data we present here are limited, they do provide some support for the idea that incentives might be reasonably opposed on the basis that they may corrupt a contribution with social, rather than monetary value. It is, however, worth considering the difference between a small token, such as a free coffee (which might be viewed as a ‘thank you’ for participation) and large financial incentives: in our study, some participants explicitly stated that small incentives would be more acceptable than large ones.

It is important not to over‐simplify the views that participants in our study expressed with regard to issues surrounding choice to participate in the testing programme. While a sense of obligation to protect others in the community was a strong feature of many accounts, some participants demonstrated more individualistic, rights‐based reasoning about choice to participate in a testing programme. For these participants, choice was presented as an inalienable right of individuals, and for a few, there was an emphasis on the entitlements of individual students as customers who pay tuition fees. The concept of restricting access to educational opportunities—such as face‐to‐face teaching—for non‐participating students was strongly opposed by some, particularly those arguing on the basis of rights and entitlement. The salience of these cultural norms of individual rights may be important to the legitimacy of testing programmes, and add weight to the argument that coercive policies—particularly those based on compulsion or penalties—should be approached with caution.

A number of practical implications follow, particularly in relation to the role of communication and education as a way of supporting participation while maintaining choice. Approaches to encouraging participation should be aimed at making testing a reasonable choice for individuals—one that is grounded in solidarity and reciprocal obligations of community, while acknowledging the current limitations of the evidence on whether mass asymptomatic testing works. In highlighting the need for regular communication that promotes positive behaviours and emphasizes the desirability of adhering to public health guidance, our study is consistent with other research examining student attitudes to COVID‐19 public health measures.^37^ There are also parallels between our findings and the existing literature surrounding vaccination. Recent work considering approaches to encouraging COVID‐19 vaccination has emphasized that persuasion may be the best strategy to encourage uptake,^38^ although it is important to note that others have advocated for a more coercive approach.^39^ Previously, research into influenza vaccination demonstrated that building trust through communications can encourage uptake.^40^ The usefulness of utilizing loss‐framed messaging—emphasizing the negative consequences of not being vaccinated—has been explored,^41^ while other evidence suggests that communicating the social benefit of vaccination may be more effective than emphasizing individual benefit in encouraging uptake.^42^ Given that students seem to be more concerned with the asymptomatic spread of COVID‐19 to others than the risk of contracting it themselves,^43^ such an approach might be helpful in encouraging participation in a student testing programme. It also suggests a challenge to the current formulation of the intervention ladder (Figure 1), which places measures such as providing information to enable choice on the rung above doing nothing, but below the use of incentives. As Owens and Cribb point out, the current intervention ladder model implies that providing information infringes upon liberty, when in fact it can promote autonomous decision‐making.^44^

This study has a number of limitations. We collected data at a particular time from a particular institution, in a rapidly‐changing pandemic. Results should be interpreted in this context. At the time our study was undertaken, participation in the testing programme was high, and the results—and conclusions drawn—might have been different if it had been lower. The collegiate nature of the University of Cambridge, which is relatively uncommon in UK higher education institutions, may have influenced some of our findings, for example those relating to what those within small communities owe to each other. The programme we studied used nasal swab‐based PCR testing and was based on a pooled testing approach, so the generalizability of our data to programmes using other technologies (e.g. lateral flow tests) is unclear. Potential issues with recruitment and sampling (e.g. bias in the stakeholders who engaged with the study) must be considered; we were unable to assess patterns of non‐response to our study, and it is possible that our findings are not representative of the views of all students and staff. Differences in the number of participants in student versus staff subgroups precluded statistical analysis for significant differences in their views in the quantitative data. Our study would benefit from replication across a wider variety of student populations in other higher education populations, in particular to clarify the generalizability of our findings.

## 5 CONCLUSION

This study provides empirical support for the principle of the least restrictive means in the context of encouraging participation in an asymptomatic student COVID‐19 testing programme: the use of policies that support choice (such as providing information about the testing programme and supporting evidence) should be used in preference to those that restrict choice (such as making participation mandatory or penalizing those who do not participate). Education and communication that outlines potential community benefits to testing and emphasizes solidarity, while enabling choice and respecting students as moral agents, may be the most optimal strategy for an effective and ethically sound asymptomatic student COVID‐19 testing programme. Further evaluation of these conclusions in different populations, and as the pandemic evolves, would be of benefit.

## AUTHOR CONTRIBUTIONS

Mary Dixon‐Woods and Caitríona Cox conceived of the study. Caitríona Cox, Mary Dixon‐Woods, Akbar Ansari, Meredith McLaughlin and Jan W. van der Scheer designed the study and developed the methods. Caitríona Cox, Akbar Ansari and Meredith McLaughlin undertook data collection. Jenny George, Jennifer Bousfield, Brandi Leach and Sarah Parkinson assisted with coding of qualitative data, and with quantitative analysis and visualization of data. Caitríona Cox, Akbar Ansari and Meredith McLaughlin analysed and interpreted the data. Caitríona Cox produced the first draft of the paper, with close support from Mary Dixon‐Woods; together they engaged in ethical analysis and developed the arguments. The final paper is the result of their collaboration. All authors reviewed the final draft.

## CONFLICT OF INTEREST

The authors declare no conflict of interest.

### DATA AVAILABILITY STATEMENT

The study instruments (interview guide and questionnaire) are available:

Repository: Guiding organisational decision‐making about Covid‐19 asymptomatic testing in higher education institutions: mixed‐method study to inform an ethical framework. https://doi.org/10.17605/OSF.IO/6VS2D.

This project contains the following files:

- Supporting Information Appendix S1—Survey.
- Supporting Information Appendix S2—Interview guide.

Owing to the conditions of the ethical approval for the project, the raw data (transcripts and survey responses) are not available for deposit. This is owing to the sensitive nature of the responses, including their possible political nature, and concerns that it would be difficult to completely de‐identify participants (who often gave extensive and specific details about their college and own circumstances in answering questions).

Any requests for access to or use of the data should be made to director@thisinstitute.cam.ac.uk. Access to fully anonymized data for suitable purposes may be granted to bona fide researchers under a data sharing agreement, but must be approved by the relevant ethics committee.

## FUNDING AND ETHICS STATEMENT

Caitríona Cox, lead researcher, is a National Institute for Health Research (NIHR) academic clinical fellow. This study is funded by Mary Dixon‐Woods’ NIHR Senior Investigator award (NF‐SI‐0617‐10026), by the Wellcome Trust through a contract award for a project on ethical issues in COVID‐19 testing, and by The Healthcare Improvement Studies Institute (THIS Institute), University of Cambridge. THIS Institute is supported by the Health Foundation, an independent charity committed to bringing about better health and healthcare for people in the UK.

The views expressed in this article are those of the authors and not necessarily those of the NHS, the NIHR, or the Department of Health and Social Care or the Wellcome Trust. The study received approval from the University of Cambridge Psychology Research Ethics Committee.

## Footnotes

1 Buchanan, D. R. (2008). Autonomy, paternalism, and justice: Ethical priorities in public health. *American Journal of Public Health, 98*(1), 15–21. https://www.ncbi.nlm.nih.gov/pmc/articles/PMC2156058/pdf/0980015.pdf; Gostin, L. O., Gravely, S. D., Shakman, S., & Markel, H. (2004). Quarantine: Voluntary or not? *The Journal of Law, Medicine & Ethics, 32*(Suppl 4), 83–86; Lee, L. (2012). Public health ethics theory: Review and path to convergence. *Journal of Law, Medicine and Ethics, 40*(1), 85–98; Swain, G. R., Burns, K. A., & Etkind, P. (2008). Preparedness: Medical ethics versus public health ethics. *Journal of Public Health Management and Practice, 14*(4), 354–357; Thomas, J. C., Sage, M., Dillenberg, J., & Guillory, V. J. (2002). A code of ethics for public health. *American Journal of Public Health, 92*(7), 1057–1059.

2 Childress, J. F., Faden, R. R., Gaare, R. D., Gostin, L. O., Kahn, J., Bonnie, R. J., Kass, N. E., Mastroianni, A. C., Moreno, J. D., & Nieburg, P. (2002). Public health ethics: Mapping the terrain. *The Journal of Law, Medicine & Ethics, 30*(2), 170–178. https://doi.org/10.1111/j.1748-720X.2002.tb00384.x; Kass, N. E. (2001). An ethics framework for public health. *American Journal of Public Health, 91*(11), 1776–1182. https://www.ncbi.nlm.nih.gov/pmc/articles/PMC1446875/pdf/0911776.pdf; Marckmann, G., Schmidt, H., Sofaer, N., & Strech, D. (2015). Putting public health ethics into practice: A systematic framework. *Frontiers in Public Health, 3*, 23. https://doi.org/10.3389/fpubh.2015.00023; Upshur, R. E. G. (2002). Principles for the justification of public health intervention. *Canadian Journal of Public Health, 93*(2), 101–103. https://www.ncbi.nlm.nih.gov/pmc/articles/PMC6979585/pdf/41997_2002_Article_BF03404547.pdf

3 Nuffield Council on Bioethics. (2007). *Public health: Ethical issues*. Nuffield Council on Bioethics.

4 Smith, C. B., Battin, M. P., Jacobson, J. A., & Francis, L. P. (2004). Are there characteristics of infectious diseases that raise special ethical issues? *Developing World Bioethics, 4*(1), 1–16.

5 Buchanan, op. cit. note 1.

6 Upshur, op. cit. note 2.

7 Gostin, L. (2006). Public health strategies for pandemic influenza: Ethics and the law. *JAMA, 295*(14), 1700–1704.

8 Raffle, A. E., & Gill, M. (2021). Mass screening for asymptomatic SARS‐CoV‐2 infection. *BMJ, 373*, n1058.

9 Raffles, A. E., & Gill, M. (2020). Screening the healthy population for covid‐19 is of unknown value, but is being introduced nationwide. *BMJ, 371*, m4438. https://doi.org/10.1136/bmj.m4438.

10 Iacobucci, G. (2020). Covid‐19: Government faces criticism over £500m plan to pilot mass testing. *BMJ, 370*, m3482.

11 Mahase, E. (2020). Covid‐19: Mass testing is inaccurate and gives false sense of security, minister admits. *BMJ, 371*, m4916. https://doi.org/10.1136/bmj.m4916; Mahase, E. (2021). Covid‐19: People are not being warned about pitfalls of mass testing. *BMJ, 372*, n238.

12 Iacobucci, G. (2021). Covid‐19: Mass testing at UK universities is haphazard and unscientific, finds BMJ investigation. *BMJ, 372*, n848.

13 Department of Education. (2021). Higher education: Reopening buildings and campuses. Retrieved April 20, 2021, from https://www.gov.uk/government/publications/higher-education-reopening-buildings-and-campuses/higher-education-reopening-buildings-and-campuses#compliance-with-guidance

14 Ives, J., Dunn, M., Molewijk, B., Schildmann, J., Bærøe, K., Frith, L., Huxtable, R., Landeweer, E., Mertz, M., Provoost, V., Rid, A., Salloch, S., Sheehan, M., Strech, D., de Vries, M., & Widdershoven, G. (2018). Standards of practice in empirical bioethics research: Towards a consensus. *BMC Medical Ethics, 19*(1), 68; McWhirter, R. E., Critchley, C. R., Nicol, D., Chalmers, D., Whitton, T., Otlowski, M., Burgess, M. M., & Dickinson, J. L. (2014). Community engagement for big epidemiology: Deliberative democracy as a tool. *Journal of Personalized Medicine, 4*(4), 459–474.

15 Kass, op. cit. note 2; Marckmann et al., op. cit. note 2.

16 Cox, C., Ansari, A., McLaughlin, M., & Van der Scheer, J. (2021). Developing an ethical framework for asymptomatic COVID‐19 testing programmes in higher education institutions. *Wellcome Open Research, 6*, 101.

17 THIS Institute. (2020). *Testing times: An ethical framework and practical recommendations for COVID‐19 testing for NHS workers*.

18 Malterud, K., Siersma, V. D., & Guassora, A. D. (2016). Sample size in qualitative interview studies: Guided by information power. *Qualitative Health Research, 26*(13), 1753–1760.

19 Creswell, J. W. (2013). Advanced mixed methods research designs. In A. Tashakkori & C. Teddlie (Eds.), *Handbook of mixed methods in social and behavioral research* (pp. 209–240). SAGE Publications.

20 Blum, J. D., & Talib, N. (2006). Balancing individual rights versus collective good in public health enforcement. *Medicine and Law, 25*, 273–281; Buchanan, op. cit. note 1.

21 Owens, J., & Cribb, A. (2013). Beyond choice and individualism: Understanding autonomy for public health ethics. *Public Health Ethics, 6*(3)262–271; Zimmerman, F. J. (2017). Public health autonomy: A critical reappraisal. *Hastings Center Report, 47*(6), 38–45.

22 Blum & Talib, op. cit. note 21.

23 Childress et al., op. cit. note 2; Kass, op. cit. note 2.

24 Nuffield Council on Bioethics, op. cit. note 3.

25 Nuffield Council on Bioethics, op. cit. note 3; Upshur, op. cit. note 2.

26 Childress et al., op. cit. note 2.

27 Cox et al., op. cit. note 16.

28 (2021). *Genomic epidemiology of SARS‐CoV‐2 in the University of Cambridge identifies dynamics of transmission: an interim report 2021*. Retrieved April 20, 2021, from https://www.gov.uk/government/publications/genomic-epidemiology-of-sars-cov-2-in-the-university-of-cambridge-identifies-dynamics-of-transmission-an-interim-report-10-december-2020

29 Nuffield Council on Bioethics, op. cit. note 3.

30 Byskov, M. F. (2019). Qualitative and quantitative interpretations of the least restrictive means. *Bioethics, 33*(4), 511–521.

31 Blake, H., Knight, H., Jia, R. Corner, J., Morling, J. R., Denning, C., Ball, J. K., Bolton, K., Figueredo, G., Morris, D. E., Tighe, P., Villalon, A. M., Ayling, K., & Vedhara, K. (2021). Students' views towards SARS‐CoV‐2 mass asymptomatic testing, social distancing and self‐isolation in a university setting during the COVID‐19 pandemic: A qualitative study. *International Journal of Environmental Research and Public Health, 18*(8), 4182.

32 Childress et al., op. cit. note 2.

33 Brownsword, R. (2005). Code, control, and choice: Why East is East and West is West. *Legal Studies, 25*(1), 1–21.

34 Buchanan, op. cit. note 1.

35 Sandel, M. J. (2012). *What money can't buy: The moral limits of markets*. Macmillan.

36 Brown, R. C. H. (2017). Social values and the corruption argument against financial incentives for healthy behaviour. *Journal of Medical Ethics, 43*(3)140–144.

37 Blake et al., op. cit. note 32.

38 Pennings, S., & Symons, X. (2021). Persuasion, not coercion or incentivisation, is the best means of promoting COVID‐19 vaccination. *Journal of Medical Ethics, 47*(10), 709–711.

39 Savulescu, J. (2021). Good reasons to vaccinate: Mandatory or payment for risk? *Journal of Medical Ethics, 47*(2), 78–85.

40 Prematunge, C., Corace, K., McCarthy, A., Nair, R. C., Pugsley, R., & Garber, G. (2012). Factors influencing pandemic influenza vaccination of healthcare workers—A systematic review. *Vaccine, 30*(32), 4733–4743. https://www.sciencedirect.com/science/article/pii/S0264410X12007190?via%3Dihub; Stead, M., Critchlow, N., Patel, R., MacKintosh, A. M., & Sullivan, F. (2019). Improving uptake of seasonal influenza vaccination by healthcare workers: Implementation differences between higher and lower uptake NHS trusts in England. *Infection, Disease & Health, 24*(1), 3–12.

41 Nan, X., Xie, B., & Madden, K. (2012). Acceptability of the H1N1 vaccine among older adults: The interplay of message framing and perceived vaccine safety and efficacy. *Health Communication, 27*(6), 559–568.

42 Betsch, C., Böhm, R., & Korn, L. (2013). Inviting free‐riders or appealing to prosocial behavior? Game‐theoretical reflections on communicating herd immunity in vaccine advocacy. *Health Psychology, 32*(9), 978–985.

43 Blake et al., op. cit. note 32.

44 Owens & Cribb, op. cit. note 22.

